# Selective Kv1.3 inhibition promotes M2 microglial polarization, accelerates hematoma resolution, and improves neurological recovery following collagenase-induced spontaneous intracerebral hemorrhage in mice

**DOI:** 10.14202/vetworld.2026.3474-3497

**Published:** 2026-08-11

**Authors:** Wachirapong Saleeon, Anchalee Vattarakorn, Namphung Thongta, Yingrak Boondam, Aree Wanasuntronwong, Wattana B. Watanapa, Sompol Tapechum, Narawut Pakaprot

**Affiliations:** 1 Department of Physiology, Faculty of Medicine Siriraj Hospital, Mahidol University, Bangkok 10700, Thailand.; 2 Department of Physiology, Faculty of Pharmacy, Mahidol University, Bangkok 10400, Thailand.; 3 Department of Oral Biology, Faculty of Dentistry, Mahidol University, Bangkok 10400, Thailand.

**Keywords:** collagenase-induced intracerebral hemorrhage, hemorrhagic stroke, Kv1.3, microglia polarization, neuro-inflammation, neuroprotection, PAP-1, spontaneous intracerebral hemorrhage

## Abstract

**Background and Aim:**

Spontaneous intracerebral hemorrhage (sICH) is a devastating subtype of stroke characterized by high mortality, severe neurological disability, and limited effective pharmacological treatment. Secondary brain injury following sICH is largely driven by excessive neuroinflammation, in which activated microglia predominantly adopt a pro-inflammatory (M1-like) phenotype that exacerbates neuronal damage. The voltage-gated potassium channel Kv1.3 is highly expressed in activated microglia and represents a promising therapeutic target for modulating neuroinflammatory responses. This study investigated whether selective Kv1.3 inhibition with 5-(4-phenoxybutoxy) psoralen (PAP-1) could promote immuno-modulatory microglial polarization, attenuate brain injury, and improve neurological recovery in a collagenase-induced mouse model of sICH.

**Materials and Methods:**

Adult male ICR mice with collagenase-induced sICH received daily intraperitoneal administration of PAP-1 (40 mg/kg) or vehicle for up to 7 days. Survival, body weight, hematoma volume, neuronal survival, Kv1.3 expression, and microglial polarization were evaluated using histological and immunohistochemical analyses. Neurological recovery was assessed using the modified neurological severity score, cylinder test, corner turn test, and open field test. Histological analyses were performed on post-injury days 1, 2, 3, and 7, whereas behavioral assessments and survival monitoring were conducted longitudinally throughout the experimental period.

**Results:**

Selective Kv1.3 inhibition significantly improved neurological recovery following sICH. PAP-1 treatment increased 7-day survival from 40% to 70% and significantly reduced hematoma volume from day 2 onward, with reductions of approximately 19% on days 2 and 3 and 24% on day 7 compared with vehicle-treated mice. PAP-1 preserved perihematomal neurons, producing an approximately 2.9-fold increase in NeuN-positive neuronal density on day 7. Furthermore, PAP-1 suppressed Kv1.3 expression, reduced CD16/32-positive M1-like microglia, enhanced CD206-positive M2-like microglial polarization beginning on day 1, increased the M2/M1 ratio, and significantly improved sensorimotor and locomotor performance across all behavioral assessments. These findings demonstrate that selective Kv1.3 inhibition effectively attenuated neuroinflammation, accelerated hematoma resolution, and promoted functional recovery after experimental sICH.

**Conclusion:**

Selective inhibition of Kv1.3 with PAP-1 promoted a favorable shift toward an immunomodulatory microglial phenotype, reduced hematoma burden, preserved perihematomal neurons, and accelerated neurological recovery following collagenase-induced sICH. The integration of histological, immunological, and functional outcomes provides strong evidence supporting Kv1.3 as a promising therapeutic target for early intervention after hemorrhagic stroke. Although the study was limited to male mice, was restricted to a 7-day observation period, and lacked detailed downstream mechanistic analyses, the findings establish a robust preclinical foundation for future investigations into long-term functional recovery, molecular signaling pathways, and translational evaluation of Kv1.3-targeted therapies in sICH.

## INTRODUCTION

Spontaneous intracerebral hemorrhage (sICH) is a severe subtype of stroke caused by the rupture of cerebral blood vessels in the absence of trauma, resulting in the direct extravasation of blood into the brain parenchyma. Hypertension is the most common underlying cause, followed by cerebral amyloid angiopathy, vascular malformations, and coagulopathies. Although sICH accounts for only 10%–15% of all stroke cases, the mortality rate approaches 45%, and more than one-third of survivors experience severe, long-term neurological disability [1]. These poor clinical outcomes reflect the combined effects of primary and secondary brain injury. Primary injury occurs within minutes to hours after hemorrhage, when the expanding hematoma mechanically compresses adjacent brain tissue, disrupts neuronal connectivity, and elevates intracranial pressure [2, 3]. This initial insult is followed by a complex secondary injury cascade triggered by hematoma degradation and the release of toxic hemoglobin breakdown products, primarily heme and iron, leading to oxidative stress, lipid peroxidation, and neuroinflammation. In addition, damage-associated molecular patterns released from necrotic cells amplify microglial activation [4] and leukocyte infiltration, promoting blood–brain barrier (BBB) disruption, cerebral edema, and ultimately delayed neuronal death [5–7].

Microglia, the resident immune cells of the central nervous system (CNS), play a central role in orchestrating post-sICH neuroinflammation. During the acute phase, activated microglia predominantly polarize toward the pro-inflammatory M1-like phenotype, producing inflammatory mediators such as tumor necrosis factor-alpha (TNF-α), interleukin (IL)-1β, and inducible nitric oxide synthase (iNOS)-derived reactive oxygen species (ROS), which collectively exacerbate oxidative stress and neuronal injury [8]. Conversely, immunomodulatory M2-like microglia secrete neurotrophic and reparative factors, including IL-10, arginase-1 (Arg1), brain-derived neurotrophic factor, and glial cell line-derived neurotrophic factor, thereby facilitating tissue repair, hematoma clearance, and neurological recovery [9, 10]. However, the early post-sICH period is characterized by predominant M1-like polarization, which substantially contributes to secondary brain injury, highlighting the therapeutic potential of interventions that promote a timely transition toward the reparative M2-like phenotype [11–13].

The voltage-gated potassium channel Kv1.3 is selectively upregulated in activated microglia and plays a critical role in sustaining pro-inflammatory activity by regulating membrane potential, calcium signaling, cytokine release, and ROS production. Genetic deletion of Kv1.3 or pharmacological inhibition with selective inhibitors, such as PAP-1 (5-(4-phenoxybutoxy) psoralen), has been shown to suppress M1-like polarization while preserving or enhancing M2-like microglial functions [14, 15]. Consequently, Kv1.3 inhibition attenuates neuroinflammation, reduces tissue injury, and improves functional recovery in experimental models of ischemic stroke and other neurodegenerative disorders [16, 17]. PAP-1 is a highly selective small molecule Kv1.3 inhibitor with demonstrated BBB permeability and immunomodulatory activity in murine models of neuroinflammation [18]. The 40 mg/kg dose used in the present study was selected based on previous investigations demonstrating effective Kv1.3 inhibition and neuroprotective efficacy in experimental CNS injury models [19, 20].

Recent evidence has demonstrated that Kv1.3 inhibition using the autologous blood injection model of intracerebral hemorrhage (ICH) alleviates white matter injury by modulating M1/M2 microglial polarization through the nuclear factor-kappa B (NF-κB) and p50 signaling pathways [20]. Nevertheless, important differences exist between the autologous blood and collagenase-induced ICH models. The autologous blood model primarily reproduces the immediate mass effect of an established hematoma and predominantly reflects secondary injury mechanisms. In contrast, the collagenase-induced model produces progressive vascular disruption, continuous blood extravasation, hematoma expansion, and evolving neuroinflammatory responses that more closely resemble the early pathophysiological events of human sICH [2]. Consequently, the collagenase model provides a more clinically relevant experimental platform for investigating microglial activation, hematoma evolution, and therapeutic interventions targeting secondary brain injury.

Although selective Kv1.3 inhibition has demonstrated neuroprotective effects in experimental ischemic stroke, neurodegenerative diseases, and more recently in the autologous blood injection model of ICH, its therapeutic potential has not been investigated in the collagenase-induced model of sICH, which more accurately reproduces progressive vascular injury and dynamic neuroinflammatory responses observed in patients. Furthermore, it remains unclear whether pharmacological inhibition of Kv1.3 can simultaneously promote M2-like microglial polarization, accelerate hematoma resolution, preserve perihematomal neurons, and improve neurological recovery within this clinically relevant model. Addressing these knowledge gaps is essential to determine the translational potential of Kv1.3-targeted therapy as a strategy to limit secondary brain injury after sICH.

This study aimed to evaluate the therapeutic potential of the selective Kv1.3 inhibitor PAP-1 in a collagenase-induced mouse model of sICH by determining whether pharmacological Kv1.3 inhibition modulates microglial/macrophage polarization, reduces hematoma burden, preserves perihematomal neuronal populations, and improves neurological recovery. We hypothesized that systemic administration of PAP-1 would (i) improve post-sICH survival and functional recovery, (ii) reduce hematoma volume and neuronal loss within the perihematomal region, and (iii) promote a shift in microglial polarization toward the immunomodulatory M2-like phenotype. To test these hypotheses, survival, body weight, histopathological alterations, Kv1.3 expression, microglial phenotype balance (CD16/32-positive M1-like and CD206-positive M2-like microglia), and neurological outcomes were evaluated during the acute and subacute phases (days 1–7) following collagenase-induced sICH. By integrating histopathological, immunohistochemical, and functional assessments, this study sought to clarify the role of Kv1.3 in the pathophysiology of sICH and to evaluate its potential as a therapeutic target to mitigate secondary brain injury. Because only male mice were included in this initial mechanistic investigation to minimize variability due to sex hormone fluctuations, extrapolation of the findings to female animals requires further validation.

## MATERIALS AND METHODS

### Ethical approval

All experimental procedures involving animals were reviewed and approved by the Animal Ethics Committee of the Faculty of Medicine Siriraj Hospital, Mahidol University, Bangkok, Thailand (Approval No. SI-ACUP 007/2564). The study was conducted in accordance with the institutional and national guidelines governing the care and use of laboratory animals and was reported in compliance with the Animal Research: Reporting of *In Vivo* Experiments 2.0 (ARRIVE) guidelines.

All surgical and experimental procedures were performed by trained personnel under controlled laboratory conditions. Mice were anesthetized before stereotaxic intracerebral injection, and their body temperature was maintained throughout surgery. Animals were continuously monitored during postoperative recovery and throughout the experimental period for changes in body weight, neurological status, general condition, and signs of treatment-related adverse effects.

The experimental design incorporated random allocation, predefined inclusion and exclusion criteria, blinded behavioral assessment, and sample size determination to minimize bias and avoid unnecessary animal use. The number of animals was limited to the minimum required to achieve the scientific objectives, and all reasonable measures were taken to minimize pain, distress, and suffering. Humane handling, appropriate anesthesia, careful postoperative observation, and standardized experimental procedures were applied in accordance with the principles of replacement, reduction, and refinement. Animals assigned to terminal histopathological and immunohistochemical assessments were euthanized at predetermined experimental time points before brain tissue collection.

### Study period and location

The study was conducted from September 10, 2021, to November 6, 2024, at the Faculty of Medicine Siriraj Hospital, Mahidol University, Bangkok, Thailand. Animal housing and acclimatization were performed at the Siriraj Laboratory Animal Research and Care Center, Faculty of Medicine Siriraj Hospital, Mahidol University, while all surgical procedures, behavioral assessments, histopathological examinations, immunohistochemical analyses, and data collection were carried out in the associated research laboratories of the Faculty of Medicine Siriraj Hospital.

### Study design

This randomized controlled experimental study was designed to evaluate the neuroprotective effects of PAP-1, a selective Kv1.3 inhibitor, in a collagenase-induced mouse model of sICH. Animals were randomly allocated to three experimental groups before surgical procedures and treatment administration: Sham + vehicle, ICH + vehicle, and ICH + PAP-1. Two independent cohorts of ICR mice were used to evaluate functional and histopathological outcomes.

### Animals

Male ICR mice (6–8 weeks of age; body weight, 32.58 ± 0.40 g) were obtained from the National Laboratory Animal Center, Mahidol University, Nakhon Pathom, Thailand, and acclimatized at the Siriraj Laboratory Animal Research and Care Center, Faculty of Medicine Siriraj Hospital, Mahidol University, Bangkok, Thailand, before the initiation of experimental procedures. Animals were housed under standard laboratory conditions at an ambient temperature of 22°C–25°C, relative humidity of 55%–60%, and a 12-h light/12-h dark cycle, with unrestricted access to standard laboratory chow and drinking water throughout the study.

### Experimental design

Animals from both cohorts were randomly assigned to one of three experimental groups using the built-in randomization function in Microsoft Excel version 16 (Microsoft Corporation, Redmond, WA, USA): Sham + vehicle, ICH + vehicle, and ICH + PAP-1.

Cohort 1 was used to evaluate survival, body weight, and neurological function. Survival was monitored daily through day 7 following sICH induction or Sham surgery. Body weight was recorded daily at approximately the same time from the pre-ICH baseline (day 0) through day 7 (n = 10 per group). Neurological performance was assessed using the modified neurological severity score (mNSS), open field, corner turn, and cylinder tests at baseline and on post-ICH days 1 (24 h), 2, 3, and 7. For consistency in data presentation, body weight comparisons are reported for baseline (day 0) and post-ICH days 1, 2, 3, and 7.

Cohort 2 was used for histopathological and immunohistochemical analyses. Animals were euthanized on post-ICH days 1 (24 h), 2, 3, and 7 (n = 6 per treatment group at each time point), and brain tissues were collected for subsequent analyses.

In both cohorts, mice in the Sham + vehicle group received an intracerebral injection of 0.5 µL sterile 0.9% saline. Mice in the ICH + vehicle group received a 0.5 µL intracerebral collagenase injection for sICH induction followed by daily intraperitoneal administration of vehicle consisting of 5% dimethyl sulfoxide (DMSO) and 95% corn oil at 10 mL/kg body weight. Mice in the ICH + PAP-1 group received identical collagenase injections followed by daily intraperitoneal administration of PAP-1 (P6124; MedChemExpress, Monmouth Junction, NJ, USA) dissolved in the same vehicle at 10 mL/kg body weight.

**Figure 1:**
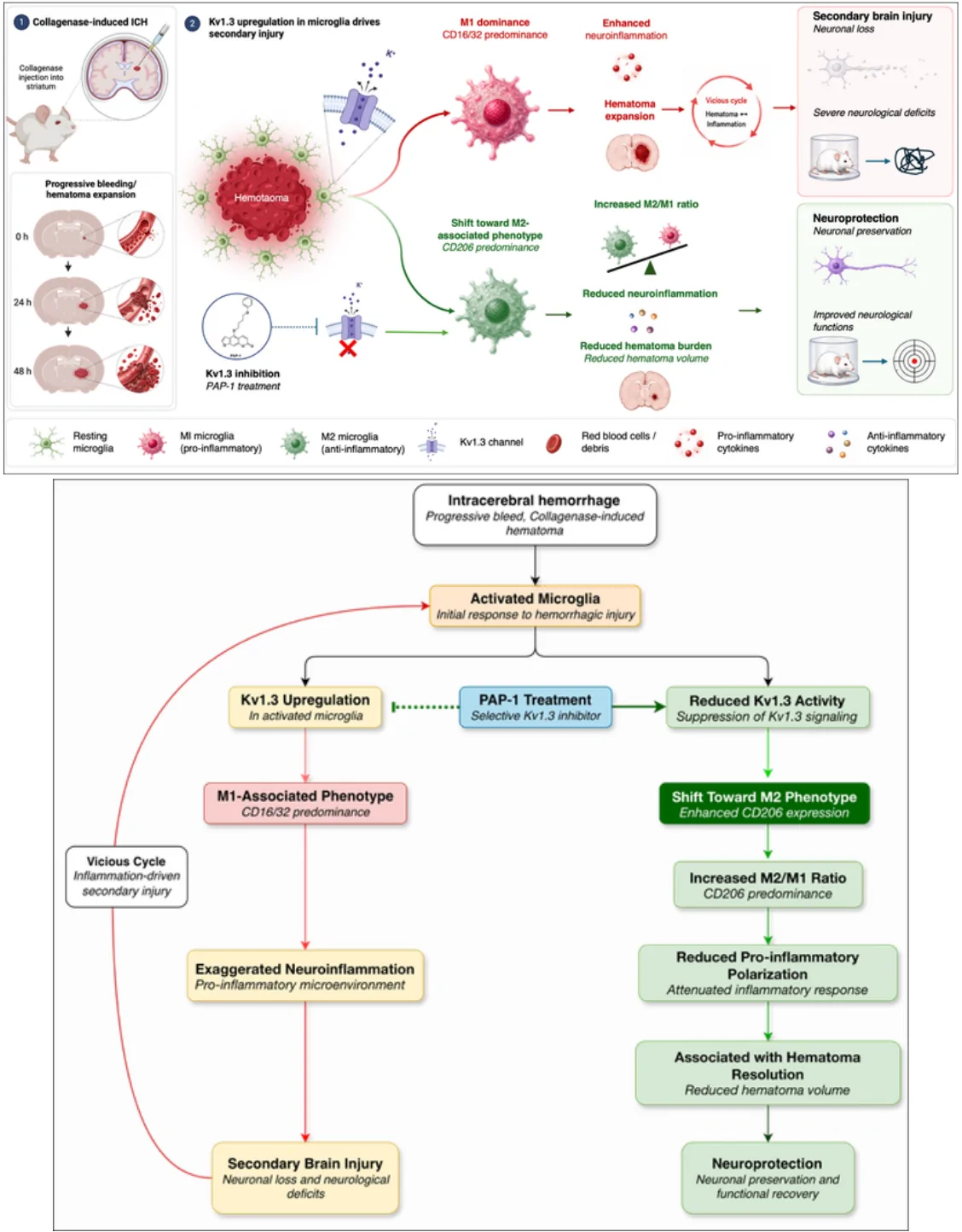
Overview of the experimental workflow and the proposed mechanism of PAP-1-mediated neuroprotection following collagenase-induced intracerebral hemorrhage. Progressive hematoma expansion induces Kv1.3 upregulation in activated microglia, promoting M1-dominant neuroinflammation and secondary injury. Pharmacological inhibition of Kv1.3 by PAP-1 suppresses pro-inflammatory signaling, promotes rapid M2 polarization, enhances hematoma clearance, and preserves neuronal survival.

**Figure 2:**
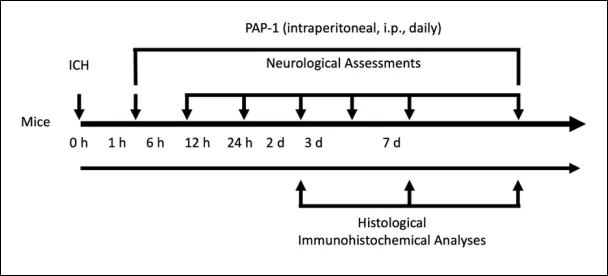
Experimental design schematic. Two independent mouse cohorts were randomly allocated to three experimental groups: Sham + vehicle, collagenase-induced intracerebral hemorrhage (ICH) + vehicle, and ICH + PAP-1. The ICH model was established by a single intracerebral collagenase injection, whereas Sham-operated mice received an equal volume of vehicle. Animals subsequently received daily intraperitoneal (i.p.) administration of PAP-1 or vehicle. Cohort 1 (n = 10/group) was used to evaluate survival through Day 7, daily body weight from baseline to Day 7, and neurological function using the mNSS, cylinder, corner turn, and open field tests at baseline and post-ICH Days 1 (24 h), 2, 3, and 7. Cohort 2 (n = 6/group/time point) was used for histopathological and immunohistochemical analyses on post-ICH Days 1 (24 h), 2, 3, and 7. ICH = Intracerebral hemorrhage; i.p. = Intraperitoneal; mNSS = Modified neurological severity score.

All animals were assigned unique identification codes, and experimental data were recorded electronically using Microsoft Excel. Sample size was calculated using G*Power version 3.1 (Heinrich Heine University Düsseldorf, Düsseldorf, Germany). Based on an expected effect size of 0.5, a significance level (α) of 0.05, and 80% statistical power, 10 animals per group were required for Cohort 1 and six animals per treatment group at each time point for Cohort 2. The study design incorporated longitudinal behavioral assessments together with multi-regional histopathological and immunohistochemical analyses at multiple acute and subacute time points to comprehensively characterize the temporal and spatial effects of PAP-1 treatment following sICH.. An overview of the experimental workflow and the proposed mechanism of PAP-1 mediated neuroprotection following collagenase-induced intracerebral hemorrhage is presented in Figure 1. Following collagenase-induced ICH, mice were assigned to the Sham + vehicle, ICH + vehicle, or ICH + PAP-1 groups. PAP-1 treatment was designed to inhibit Kv1.3 signaling in activated microglia, thereby reducing M1-associated neuroinflammation, promoting M2-associated polarization, facilitating hematoma resolution, and improving neurological recovery. The detailed experimental design, treatment schedule, behavioral assessments, and tissue collection timeline are illustrated in Figure 2.

### ICH induction

Mice were anesthetized with a single intraperitoneal injection of ketamine (100 mg/kg) and xylazine (10 mg/kg), positioned in a stereotaxic frame, and subjected to intracerebral injection of either collagenase or vehicle (Sham group) as previously described [19]. The injection was performed into the right striatum (0.2 mm anterior to bregma, 2.0 mm lateral to the midline, and 3.7 mm below the skull surface) using a 10-µL Hamilton microsyringe (Hamilton Company, Bonaduz, Switzerland) connected to a microinfusion pump delivering the solution at a constant rate of 0.1 µL/min. The injection needle was maintained in position for 10 min after infusion to minimize reflux along the injection tract.

Type VII-S collagenase (C2399; Sigma-Aldrich, St. Louis, MO, USA) was freshly prepared in sterile saline immediately before surgery and administered at a dose of 0.075 U in 0.5 µL [21, 22]. Core body temperature was maintained between 37.0°C and 37.5°C throughout the surgical procedure using a thermostatically controlled heating pad. Animals were monitored continuously during recovery, and post-procedural body weight was recorded.

Animals were excluded if they died within 24 h after sICH induction, exhibited unsuccessful hematoma induction, or experienced technical failures during tissue processing or image acquisition. The collagenase-induced sICH model was selected because it closely reproduces progressive vascular rupture, active bleeding, hematoma expansion, and the early pathophysiological evolution of sICH observed in humans.

### Drugs and treatment regimens

The selective small molecule Kv1.3 inhibitor PAP-1 was freshly prepared immediately before administration by dissolving it in a vehicle containing 5% DMSO and 95% corn oil to ensure adequate solubility and bioavailability [23]. PAP-1 was administered intraperitoneally at a dose of 40 mg/kg once daily, beginning within 1 h after sICH induction and continuing until the predetermined experimental endpoint on day 7. Accordingly, animals assigned to terminal analyses received 1, 2, 3, or 7 daily injections before tissue collection [16, 20].

Mice in the Sham + vehicle and ICH + vehicle groups received equivalent volumes of the vehicle by intraperitoneal injection at approximately the same time each day. All injections were performed under sterile conditions, and animals were closely monitored for signs of adverse reactions throughout the treatment period. The selected PAP-1 dose was based on previous studies demonstrating effective Kv1.3 inhibition, BBB permeability, and neuroprotective efficacy, with no evidence of overt systemic toxicity [23].

### Neurological assessments

Neurological function was evaluated using a battery of behavioral tests designed to assess motor, sensory, balance, and locomotor performance at baseline (pre-ICH) and on post-ICH days 1 (24 h), 2, 3, and 7. All behavioral assessments were conducted during the light phase between 9:00 AM and 12:00 PM to minimize circadian variation. Before testing, mice were acclimatized to the behavioral testing room for at least 30 min under standardized environmental conditions. Assessments were performed under consistent ambient lighting and minimal background noise.

All behavioral tests were conducted and scored by investigators blinded to treatment allocation according to predefined evaluation criteria. For mNSS assessment, neurological scores were independently assigned by two blinded observers, and any discrepancies were resolved by consensus before statistical analysis.

### Modified neurological severity score

Neurological deficits were quantified using the mNSS, a composite neurological assessment ranging from 0 (normal neurological function) to 14 (maximum neurological impairment) [24]. The final score represented the sum of motor (0–6), sensory (0–2), and balance (0–6) subscores, with higher scores indicating greater neurological dysfunction.

Motor function was evaluated by assessing forelimb flexion, hindlimb flexion, trunk twisting or head deviation (>10°) during tail suspension, and spontaneous locomotion on a flat surface. Sensory function was assessed using visual placing and tactile/proprioceptive placing responses. Balance was evaluated using a balance beam test, with progressively higher scores assigned as impairment increased, ranging from stable balance to complete inability to maintain posture on the beam.

### Open field test

The open field test (OFT) was performed to evaluate spontaneous locomotor activity and exploratory behavior. The apparatus consisted of an opaque open-top cylindrical arena (30 cm in diameter and 40 cm in height) with a flat, non-reflective floor. Behavioral testing was conducted under constant illumination of approximately 100–150 lux. Each mouse was placed individually in the center of the arena and allowed to explore freely for 10 min [25]. The apparatus was cleaned with 70% ethanol between animals to eliminate residual olfactory cues. Total distance traveled was automatically recorded and analyzed using the Panlab SMART video tracking system (Panlab, S.L.U., Barcelona, Spain).

### Cylinder test

The cylinder test was used to evaluate forelimb use asymmetry resulting from unilateral sensorimotor deficits following sICH. Individual mice were placed in a transparent acrylic cylinder (10 cm in diameter and 15 cm in height), and spontaneous vertical exploratory behavior (rearing) was recorded. Forelimb contacts with the cylinder wall were categorized as impaired (left), unimpaired (right), or simultaneous use of both forelimbs during 20 rearing events [26, 27]. Forelimb use asymmetry was calculated as:


\[
\frac{I}{I+U+B}\times 100
\]

where I = impaired forelimb contacts, U = unimpaired forelimb contacts, and B = simultaneous contacts using both forelimbs [27].

### Corner turn test

The corner turn test was used to assess lateralized sensorimotor dysfunction following unilateral brain injury [28]. Each mouse was allowed to enter a corner formed by two boards positioned at an angle of 30°. Upon reaching the corner, the animal exited by turning either to the left or right, and the turning direction was recorded. Each animal completed 15 consecutive trials with an inter-trial interval of at least 30 s. The percentage of right (ipsilateral) turns was calculated as an index of motor asymmetry. An increased preference for ipsilateral turning indicated greater contralateral sensorimotor impairment following sICH.

### Histological and immunohistochemical analyses

**Tissue preparation:** For terminal histological and immunohistochemical analyses, mice from Cohort 2 (n = 6 per treatment group at each time point) were transcardially perfused with 1× phosphate-buffered saline (PBS; pH 7.4), followed by 4% paraformaldehyde (PFA) prepared in 0.1 M PBS. Brains were carefully harvested, post-fixed overnight in 4% PFA at 4°C, and cryoprotected in 30% sucrose solution for 48 h at 4°C until complete tissue infiltration was achieved. Serial coronal brain sections (20 µm thick) were prepared using a cryostat at five predefined anatomical levels (+2, +1, 0, −1, and −2 mm relative to the hematoma center), with the 0-mm level corresponding to the needle insertion plane.

Hematoma morphology was initially confirmed in all sections by hematoxylin and eosin (H&E) staining [29]. Adjacent serial sections from identical anatomical levels were subsequently allocated for immunohistochemical analyses of NeuN (neuronal survival), Kv1.3 (channel expression), CD16/32 (M1-like microglia), and CD206 (M2-like microglia). Negative control sections were processed in parallel by omitting the primary antibody to verify the absence of nonspecific secondary antibody staining. Primary antibodies were selected based on published validation studies and manufacturer recommendations.

Histological quantification was performed using standardized region-of-interest (ROI)-based manual cell counting rather than unbiased stereological methods. Five representative coronal sections from each animal were analyzed. Image acquisition, quantitative analyses, and cell counting were performed by a single investigator blinded to treatment allocation using predefined counting criteria.

**Histological analysis and region-of-interest definition:** Three anatomically defined ROIs were established for quantitative histological analyses: hematoma core (Zone 1), perihematomal region (Zone 2), and distant parenchyma (Zone 3). The hematoma core was manually delineated on each section using ZEN Pro software (Carl Zeiss Microscopy GmbH, Oberkochen, Germany). The perihematomal and distant parenchymal regions were subsequently generated using the ROI expansion function of the software.

Specifically, Zone 2 comprised tissue located within 0–300 µm from the hematoma boundary, whereas Zone 3 encompassed the adjacent region extending from 300 to 600 µm beyond the hematoma margin. Expansion was performed along the actual contour of the manually delineated hematoma to ensure anatomical consistency.

Five representative coronal sections (+2, +1, 0, −1, and −2 mm relative to the hematoma center) were analyzed for each animal. Quantitative measurements were obtained from standardized, non-overlapping microscopic fields under ×40 magnification. Positive cells were manually counted and averaged across all analyzed sections to generate a representative value for each animal. All analyses were performed using identical imaging parameters and anatomical landmarks across treatment groups. Although formal stereological methods were not employed, ROI-based quantification was standardized for all animals and experimental groups. Representative photomicrographs are presented, whereas quantitative analyses were performed using data obtained from all animals.

### Hematoma volume assessment

Coronal brain sections (20 µm thick) obtained at +2, +1, 0, −1, and −2 mm relative to the hematoma center were stained with H&E. Hematoma cross-sectional areas were measured using ZEN Pro software with spatial calibration. Hematoma volume was calculated according to the Cavalieri principle using the following equation:

### Hematoma volume (mm³) = Σ (Areaᵢ × Section interval)

where Areaᵢ represents the hematoma area measured in each coronal section and the section interval was 1 mm.

### Kv1.3 expression

Kv1.3 expression was evaluated by immunohistochemistry (IHC). Brain sections were washed with PBS and subjected to heat-mediated antigen retrieval in citrate buffer (pH 6.0) at 95°C for 20 min. Following cooling and PBS washing, sections were blocked with PBS containing 5% bovine serum albumin and 0.3% Triton X-100 for 1 h at 25°C before overnight incubation at 4°C with mouse monoclonal anti-Kv1.3 antibody (1:500; Santa Cruz Biotechnology, Dallas, TX, USA; Cat. No. sc-398855; RRID: AB_3094656).

After PBS washing, sections were incubated with an appropriate horseradish peroxidase (HRP)-conjugated secondary antibody for 1 h at room temperature. Immunoreactivity was visualized using 3,3′-diaminobenzidine (DAB) chromogen. Kv1.3-positive cells were quantified under ×40 magnification within the three predefined anatomical zones. Positive cells were manually counted in three standardized ROIs per zone on each section and averaged to obtain a representative value for each animal [30].

### Neuronal survival

Neuronal survival in the perihematomal region was evaluated by IHC using the mature neuronal marker NeuN. Brain sections were processed as described above and incubated overnight at 4°C with rabbit anti-NeuN primary antibody (1:500; ABclonal, Wuhan, China; Cat. No. A19086; RRID: AB_2862578), followed by HRP-conjugated secondary antibody (1:500; Abcam, Cambridge, UK). Immunoreactivity was visualized using DAB substrate (Vector Laboratories, Newark, CA, USA) and examined under bright-field microscopy.

NeuN-positive cells were manually quantified under ×40 magnification within predefined ROIs across Zones 1–3. Five representative coronal sections (+2, +1, 0, −1, and −2 mm relative to the hematoma center) were analyzed for each animal, and the mean value was calculated. Adjacent serial sections from identical anatomical levels were used for Kv1.3, CD16/32, and CD206 staining to facilitate direct regional comparisons between neuronal survival and microglial activation.

### Microglial activation and polarization

Microglial activation and polarization were assessed by IHC using CD16/32 as a marker of M1-like microglia and CD206 as a marker of M2-like microglia. Tissue sections were processed using the same protocol described for Kv1.3 and NeuN immunostaining. Briefly, sections were incubated overnight at 4°C with rat anti-CD16/CD32 antibody (1:200; Abcam, Cambridge, UK; Cat. No. ab25235; RRID: AB_470417) or mouse anti-CD206 antibody (1:200; Santa Cruz Biotechnology, Dallas, TX, USA; Cat. No. sc-58986; RRID: AB_2144945). After PBS washing, sections were incubated with HRP-conjugated secondary antibody (1:500; Abcam) for 1 h at room temperature, followed by DAB visualization.

Immunostained sections were examined under bright-field microscopy. CD16/32-positive and CD206-positive cells were manually quantified under ×40 magnification within the three predefined anatomical zones. Cell counts were obtained from three standardized ROIs per zone in each section and averaged per animal. The M2/M1 ratio (CD206/CD16/32) was subsequently calculated to assess the microglial polarization balance in response to injury and PAP-1 treatment.

### Statistical analysis

All statistical analyses were performed using GraphPad Prism version 5.0 (GraphPad Software, La Jolla, CA, USA). Data normality was assessed using the Shapiro–Wilk test before selecting parametric statistical analyses. Normally distributed data are presented as the mean ± standard error of the mean (SEM).

Depending on the experimental design, either one-way or two-way analysis of variance (ANOVA) was performed. One-way ANOVA followed by Tukey's multiple-comparison test was used to compare treatment groups at individual time points, including the following pairwise comparisons: Sham + vehicle versus ICH + vehicle, Sham + vehicle versus ICH + PAP-1, and ICH + vehicle versus ICH + PAP-1. Two-way ANOVA followed by Tukey's multiple-comparison test was used to evaluate repeated measurements and to determine the effects of treatment, time, and treatment × time interaction on longitudinal variables, including body weight and behavioral outcomes. Statistical significance was defined as p < 0.05.

Survival was analyzed using Kaplan–Meier survival curves, and differences among groups were evaluated using the log-rank (Mantel–Cox) test. Hazard ratios (HRs) with 95% confidence intervals (CIs) were estimated using the Cox proportional hazards model. Where appropriate, effect sizes (omega squared, ω²) together with 95% CIs were calculated to quantify the magnitude of treatment effects.

Sample sizes were determined on the basis of previous studies employing comparable collagenase-induced sICH models and PAP-1 treatment protocols. As sample size estimation was based on established experimental evidence, a formal *a priori* power analysis was not performed.

## RESULTS

### PAP-1 improved survival and attenuated body weight loss following ICH

**Figure 3:**
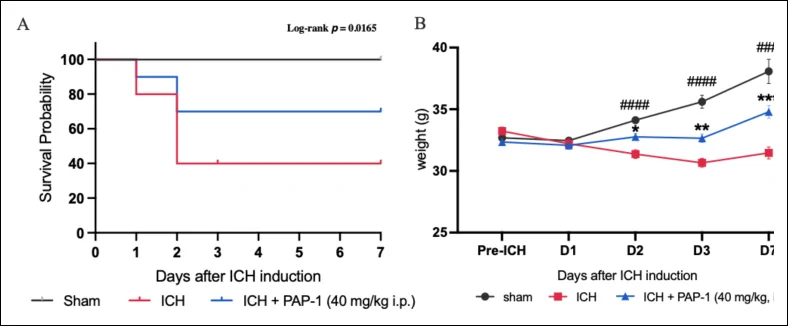
PAP-1 treatment improved survival and attenuated body weight loss following ICH induction. (A) Kaplan–Meier survival curves for the Sham + vehicle, ICH + vehicle, and ICH + PAP-1 groups (Cohort 1; n = 10 per group). Overall survival differed significantly among groups (log-rank [Mantel–Cox] test, p = 0.0165). PAP-1 treatment was associated with a lower estimated mortality risk than vehicle treatment (HR = 0.46, 95% CI = 0.12–1.85), although the pairwise comparison was not statistically significant. (B) Longitudinal changes in body weight demonstrating that PAP-1 significantly attenuated post-ICH weight loss and enhanced recovery compared with vehicle-treated mice. Data are presented as mean ± SEM. CI = Confidence interval; HR = Hazard ratio; ICH = Intracerebral hemorrhage; mNSS = Modified neurological severity score; PAP-1 = 5-(4-phenoxybutoxy) psoralen; SEM = Standard error of the mean. *p < 0.05, **p < 0.01, ***p < 0.001 versus ICH + vehicle. ###p < 0.001 and ####p < 0.0001 versus Sham + vehicle at the corresponding time points (two-way ANOVA followed by Tukey's multiple-comparison test).

Kaplan–Meier survival analysis demonstrated significant differences in survival among the experimental groups following ICH induction (log-rank [Mantel–Cox] test, p = 0.0165; Gehan–Breslow–Wilcoxon test, p = 0.0175) (Figure 3A). Although pairwise comparison between the ICH + vehicle and ICH + PAP-1 groups did not reach statistical significance, PAP-1-treated mice exhibited numerically greater survival beginning on day 2 and throughout the 7-day observation period. Cox proportional hazards analysis further demonstrated a lower estimated mortality risk in PAP-1-treated mice than in vehicle-treated ICH mice (HR = 0.46, 95% CI = 0.12–1.85); however, the confidence interval crossed unity, indicating that this comparison was not statistically significant. Final survival rates were 100% in the Sham + vehicle group, 40% in the ICH + vehicle group, and 70% in the ICH + PAP-1 group. The absence of statistical significance in the pairwise comparison may reflect the relatively small sample size.

Body weight measurements further supported the beneficial effects of PAP-1 treatment (Figure 3B). Two-way repeated-measures ANOVA demonstrated a significant treatment effect on body weight (ω² = 0.26). Body weight remained stable throughout the experimental period in the Sham + vehicle group. In contrast, both ICH groups exhibited significant body weight loss during the acute post-injury phase, with greater weight loss observed in vehicle-treated animals.

Baseline body weights did not differ significantly among the three groups (Sham + vehicle, 32.69 ± 0.39 g; ICH + vehicle, 33.23 ± 0.33 g; ICH + PAP-1, 32.08 ± 0.35 g; adjusted p = 0.5522, p = 0.7509, and p = 0.1160, respectively). Following ICH induction, mice in the ICH + vehicle group exhibited progressive weight loss, reaching 30.66 ± 0.35 g on day 3, followed by only modest recovery to 31.46 ± 0.49 g on day 7. In contrast, PAP-1-treated mice maintained body weight more effectively, with body weights of 32.75 ± 0.29 g on day 2 and 32.65 ± 0.35 g on day 3, followed by marked recovery to 34.79 ± 0.35 g on day 7.

Compared with the ICH + vehicle group, body weight was significantly greater in PAP-1-treated mice on day 2 (p = 0.0177, 95% CI = 0.2333–2.567), day 3 (p = 0.0023, 95% CI = 0.7188–3.261), and day 7 (p = 0.0004, 95% CI = 1.550–5.110). These findings indicate that PAP-1 attenuated post-ICH body weight loss and promoted physiological recovery during the acute and subacute phases following ICH.

### PAP-1 accelerated hematoma resolution and preserved perihematomal neurons

**Figure 4:**
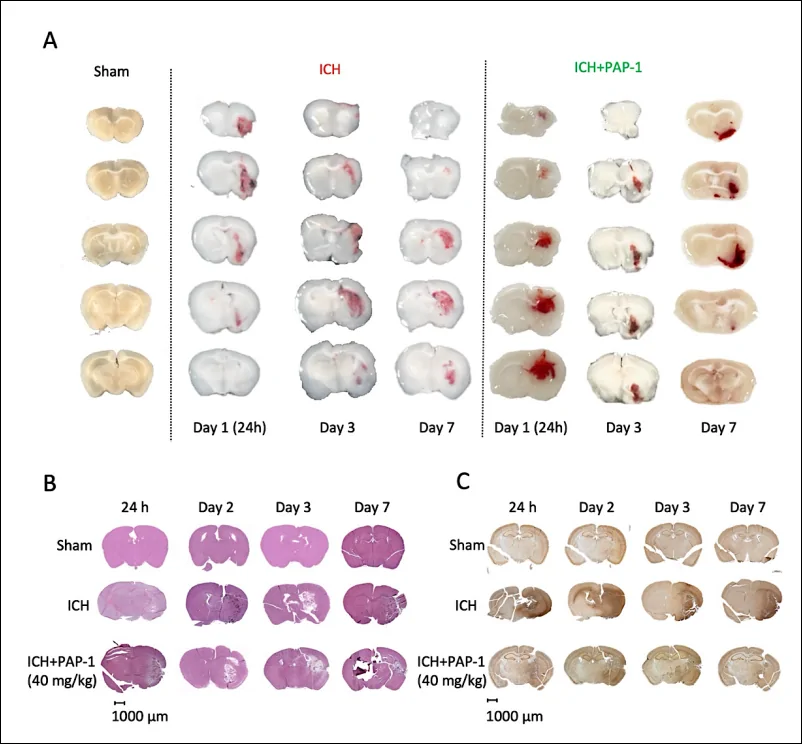
PAP-1 partially mitigated ICH induced structural brain damage and neurodegeneration. (A) Representative coronal brain sections collected on post-ICH days 1 (24 h), 3, and 7 showing progressive hematoma formation in vehicle-treated mice and improved structural preservation following PAP-1 treatment. (B) Representative H&E-stained sections illustrating the hematoma core and perihematomal regions on post-ICH days 1 (24 h), 2, 3, and 7. (C) Representative NeuN immunostaining images from the perihematomal region (Zone 2) demonstrating marked neuronal loss in vehicle-treated mice and partial neuronal preservation following PAP-1 treatment. H&E = Hematoxylin and eosin; ICH = Intracerebral hemorrhage; NeuN = Neuronal nuclei.

Representative coronal brain sections centered on the collagenase injection site demonstrated progressive neuropathological changes following ICH together with the neuroprotective effects of PAP-1 treatment (Figure 4A). Brains from the Sham + vehicle group exhibited normal gross morphology without evidence of hematoma formation. In contrast, vehicle-treated ICH mice developed progressive hematoma enlargement accompanied by marked structural disruption between 24 h and day 7 after injury. PAP-1-treated mice consistently exhibited smaller hematomas and better preservation of overall brain architecture throughout the observation period.

**Figure 5:**
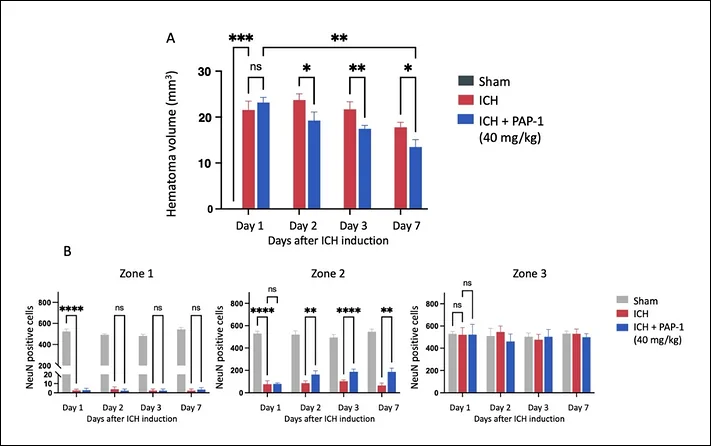
PAP-1 treatment reduced hematoma volume and enhanced neuronal survival. (A) Representative H&E-stained sections and quantitative analysis of hematoma volume demonstrating accelerated hematoma resolution and reduced lesion size in PAP-1-treated mice compared with vehicle-treated ICH mice. (B) Quantification of NeuN-positive neuronal density within Zones 1–3 demonstrating significant preservation of neurons in the perihematomal region (Zone 2) following PAP-1 treatment on post-ICH days 2, 3, and 7. Data are presented as mean ± SEM (n = 6 mice per group at each time point). H&E = Hematoxylin and eosin; ICH = Intracerebral hemorrhage; NeuN = Neuronal nuclei; PAP-1 = 5-(4-phenoxybutoxy) psoralen; SEM = Standard error of the mean; Zone 1 = Hematoma core; Zone 2 = Perihematomal region; Zone 3 = Distant parenchyma. ns = Not significant; *p < 0.05, **p < 0.01, ***p < 0.001, ****p < 0.0001.

Quantitative analysis of H&E-stained serial coronal sections (Figure 5A) demonstrated no significant difference in hematoma volume between the ICH + vehicle and ICH + PAP-1 groups on post-ICH day 1 (23.30 ± 0.44 mm³ versus 24.00 ± 0.47 mm³; p > 0.05). However, PAP-1 treatment significantly reduced hematoma volume thereafter, with a strong treatment effect (ω² = 0.95). Hematoma volume was significantly lower in the PAP-1-treated group on day 2 (19.2 ± 0.94 mm³ versus 23.7 ± 0.61 mm³; p = 0.0142, 95% CI = 0.7524–4.5960), day 3 (17.5 ± 0.38 mm³ versus 21.7 ± 0.82 mm³; p = 0.0070, 95% CI = 0.9724–5.449), and day 7 (13.5 ± 0.80 mm³ versus 17.8 ± 0.54 mm³; p = 0.0140, 95% CI = 1.157–9.110). No measurable hematoma was observed in the Sham + vehicle group at any time point (p < 0.001), confirming that needle insertion alone did not produce hemorrhagic injury.

Representative H&E-stained sections (Figure 4B) further demonstrated extensive hemorrhage and tissue distortion in vehicle-treated ICH mice, whereas PAP-1-treated animals exhibited markedly reduced hematoma size and improved preservation of tissue architecture throughout the study. These findings indicate that PAP-1 accelerated hematoma resolution and reduced hemorrhagic injury following collagenase-induced ICH.

Two-way ANOVA demonstrated a pronounced treatment effect on NeuN-positive neuronal survival within the perihematomal region (Zone 2) (ω² = 0.98, p < 0.0001). NeuN immunostaining (Figure 4C) showed substantial neuronal loss in the ICH + vehicle group compared with the Sham + vehicle group on day 7 (64.50 ± 9.33 versus 544.50 ± 9.04 cells/field, representing an approximately 88% reduction; p = 0.0001, 95% CI = −148.5 to −97.53). PAP-1 treatment significantly attenuated neuronal loss, increasing NeuN-positive cell density to 187.00 ± 11.32 cells/field, representing an approximately 2.9-fold increase compared with vehicle-treated ICH mice (p = 0.0037) (Figure 5B). In contrast, no significant differences in NeuN-positive cell counts were observed between the two ICH groups within the hematoma core (Zone 1) or the distant parenchyma (Zone 3).

Collectively, these findings demonstrate that PAP-1 promoted hematoma resolution while significantly preserving neuronal populations within the vulnerable perihematomal region following ICH.

### Kv1.3 expression and microglial polarization

**Figure 6:**
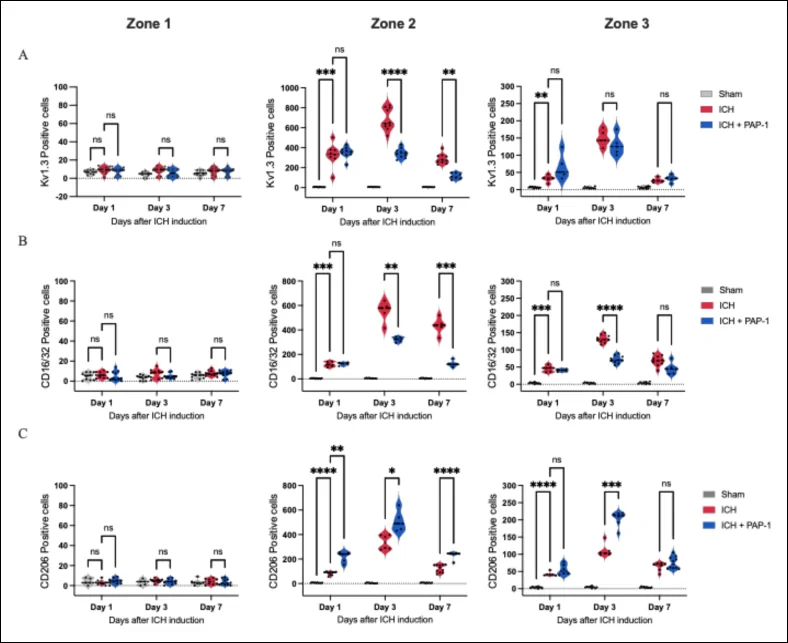
PAP-1 treatment reduced Kv1.3 expression and shifted the balance between CD16/32-positive (M1-like) and CD206-positive (M2-like) microglia toward M2 predominance. Expression levels were quantified in the hematoma core (Zone 1), perihematomal region (Zone 2), and surrounding parenchyma (Zone 3) on post-ICH days 1, 3, and 7. (A) Quantification of Kv1.3-positive cells demonstrated marked suppression in Zone 2 of PAP-1-treated mice on days 3 and 7, whereas expression remained minimal in Zones 1 and 3. (B) CD16/32-positive cells and (C) CD206-positive cells were quantified in the same regions. CD16/32 expression increased markedly in vehicle-treated mice, peaking in Zone 2 on Day 3, whereas PAP-1 significantly suppressed this response. In contrast, CD206 expression was significantly enhanced in PAP-1-treated mice, particularly within Zones 2 and 3. No substantial changes were observed in Zone 1. Results are presented as mean ± SEM (n = 6 mice per group at each time point). CI = Confidence interval; H&E = Hematoxylin and eosin; ICH = Intracerebral hemorrhage; Kv1.3 = Voltage-gated potassium channel Kv1.3; M1 = Classically activated microglia; M2 = Alternatively activated microglia; PAP-1 = 5-(4-phenoxybutoxy) psoralen; SEM = Standard error of the mean; Zone 1 = Hematoma core; Zone 2 = Perihematomal region; Zone 3 = Distant parenchyma. ns = Not significant; *p < 0.05, **p < 0.01, ***p < 0.001, ****p < 0.0001 versus ICH + vehicle (two-way ANOVA followed by Tukey's multiple-comparison test).

Immunohistochemical analysis demonstrated minimal baseline expression of Kv1.3, CD16/32, and CD206 in the Sham + vehicle group, whereas ICH induced pronounced region- and time-dependent alterations in all three markers (Figure 6). Quantitative analysis of Kv1.3, CD16/32, and CD206 immunostaining across the three anatomical regions on post-ICH days 1, 3, and 7 (Figures 6A–C) demonstrated progressive accumulation of Kv1.3-positive cells within and around the lesion.

On day 1 after ICH, Kv1.3-positive cell density was greatest in the perihematomal region (Zone 2) in both the ICH + vehicle and ICH + PAP-1 groups (328.00 ± 39.90 and 353.33 ± 18.80 cells/field, respectively), whereas substantially fewer Kv1.3-positive cells were detected in the hematoma core (Zone 1; 8.90 ± 1.23 and 7.80 ± 1.30 cells/field, respectively) and the distant parenchyma (Zone 3; 32.10 ± 4.52 and 65.40 ± 16.23 cells/field, respectively). No significant differences between the two ICH groups were observed at this time point.

By day 3, Kv1.3-positive cells reached their maximum density in Zone 2 of the ICH + vehicle group (685.00 ± 34.10 cells/field; p < 0.0001 versus Sham + vehicle). This increase was significantly attenuated by PAP-1 treatment (344.60 ± 14.96 cells/field; p < 0.01 versus ICH + vehicle; 95% CI = 276.60–404.20). Two-way ANOVA demonstrated a substantial treatment effect on Kv1.3 expression within the perihematomal region (Zone 2) (ω² = 0.64), indicating that PAP-1 markedly suppressed the post-ICH increase in Kv1.3-positive cells. In contrast, Kv1.3 expression remained consistently low in the hematoma core (Zone 1) and distant parenchyma (Zone 3) throughout the observation period. By day 7, Kv1.3-positive cell numbers in Zone 2 had declined in both treatment groups (ICH + vehicle, 288.00 ± 15.68 cells/field; ICH + PAP-1, 115.00 ± 11.10 cells/field) but remained significantly greater in vehicle-treated mice (p = 0.0022; 95% CI = 103.00–243.60). These findings indicate that Kv1.3-expressing M1-like microglia accumulated predominantly within the perihematomal region during the acute phase of collagenase-induced ICH and that pharmacological Kv1.3 inhibition effectively suppressed this inflammatory response.

Consistent with the Kv1.3 staining pattern, CD16/32-positive (M1-like) microglia accumulated progressively in vehicle-treated mice, particularly within Zones 2 and 3 (Figure 6B), whereas PAP-1 treatment significantly attenuated this pro-inflammatory response. On day 3, the density of CD16/32-positive cells in Zone 2 was significantly lower in PAP-1-treated mice than in vehicle-treated mice (322.60 ± 9.40 versus 552.60 ± 38.00 cells/field; p = 0.0046; 95% CI = 114.30–345.70). A similar reduction was observed in Zone 3, where PAP-1 treatment significantly decreased CD16/32-positive cell density compared with the ICH + vehicle group (71.80 ± 3.45 versus 131.80 ± 3.45 cells/field; p < 0.0001; 95% CI = 49.61–70.39). By day 7, CD16/32-positive cell density remained elevated in Zones 2 and 3 of the ICH + vehicle group (433.40 ± 29.70 and 68.80 ± 4.70 cells/field, respectively), whereas PAP-1-treated mice exhibited substantially lower values in Zone 2 (124.20 ± 11.41 cells/field; p = 0.0002; 95% CI = 241.40–377.00) and near baseline values in Zone 3 (45.67 ± 6.67 cells/field; p = 0.2367 versus ICH + vehicle).

Conversely, PAP-1 treatment enhanced the accumulation of CD206-positive (M2-like) microglia, particularly within the perihematomal region, indicating an earlier reparative response (Figure 6C). On day 1, significantly more CD206-positive cells were present in Zone 2 of PAP-1-treated mice than in vehicle-treated mice (217.00 ± 20.20 versus 83.80 ± 4.58 cells/field; p = 0.0071). CD206-positive cell density increased further in both groups by day 3 but remained significantly greater in the PAP-1-treated group (509.00 ± 37.96 versus 337.50 ± 24.30 cells/field; p = 0.0322 versus ICH + vehicle). A similar trend was observed in Zone 3 (204.14 ± 7.90 versus 110.50 ± 7.69 cells/field; p = 0.0007). By day 7, PAP-1-treated mice maintained significantly higher numbers of CD206-positive cells in Zone 2 (232.50 ± 12.59 versus 122.70 ± 10.16 cells/field), whereas no significant difference was observed in Zone 3 between the ICH + vehicle (65.14 ± 4.61 cells/field) and ICH + PAP-1 (75.30 ± 5.63 cells/field) groups, with both approaching baseline values.

Within the PAP-1-treated group, CD16/32-positive (M1-like) cells increased from day 1 to a transient peak on day 3 (123.00 ± 5.73 to 322.60 ± 9.40 cells/field) before declining markedly by day 7 (124.20 ± 11.41 cells/field). In contrast, CD206-positive (M2-like) cells were already significantly elevated on day 1 (217.00 ± 20.16 cells/field; p = 0.0219 versus M1-like cells), reached a maximum on day 3 (509.00 ± 37.96 cells/field; p = 0.0406 versus M1-like cells), and remained moderately elevated on day 7 (232.50 ± 12.59 cells/field; p = 0.0012 versus M1-like cells). These findings indicate that PAP-1 promoted an early shift toward an M2-like reparative microglial phenotype following ICH.

**Figure 7:**
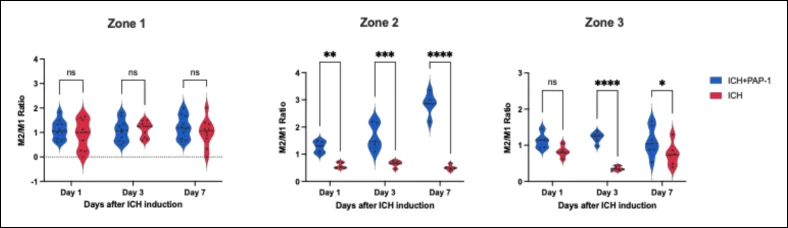
PAP-1 treatment increased the microglial M2/M1 (CD206/CD16/32) ratio in the perihematomal region following ICH. Line graphs show the M2/M1 ratio in Zones 1, 2, and 3 on post-ICH days 1, 3, and 7. Zone 1 exhibited minimal temporal variation in both treatment groups. In contrast, the PAP-1-treated group showed a progressive increase in the M2/M1 ratio in Zone 2 throughout the study, whereas the ICH + vehicle group maintained consistently lower values indicative of M1 predominance. Modest fluctuations were observed in Zone 3, although the PAP-1-treated group generally maintained higher M2/M1 ratios. Results are presented as mean ± SEM (n = 6 mice per group at each time point). ICH = Intracerebral hemorrhage; M1 = Classically activated microglia; M2 = Alternatively activated microglia; SEM = Standard error of the mean; Zone 1 = Hematoma core; Zone 2 = Perihematomal region; Zone 3 = Distant parenchyma. *p < 0.05, **p < 0.01, ***p < 0.001, ****p < 0.0001.

Two-way repeated-measures ANOVA demonstrated a substantial treatment effect on the M2/M1 microglial phenotype ratio within the perihematomal region (Zone 2) (ω² = 0.61; p = 0.0001). Consistent with the observed changes in CD16/32-positive and CD206-positive cell populations, the M2/M1 ratio (CD206/CD16/32) was significantly greater in PAP-1-treated mice than in vehicle-treated mice throughout the study (Figure 7). In the ICH + vehicle group, the M2/M1 ratio in Zone 2 remained consistently below 1.0 (Day 1, 0.58 ± 0.05; day 3, 0.65 ± 0.05; day 7, 0.51 ± 0.04). In contrast, the PAP-1-treated group maintained ratios greater than 1.0 at all time points, with progressive increases during the post-ICH period (day 1, 1.27 ± 0.10; p = 0.0030; 95% CI = 0.3109–1.069; day 3, 1.65 ± 0.22; p = 0.0003; 95% CI = 0.6244–1.382; day 7, 2.85 ± 0.19; p < 0.0001; 95% CI = 1.968–2.726). In Zone 1, the M2/M1 ratio remained relatively stable without consistent differences between treatment groups. In Zone 3, PAP-1-treated mice generally exhibited higher M2/M1 ratios than vehicle-treated mice, although only modest temporal changes were observed.

Overall, these findings demonstrate that PAP-1 shifted microglial polarization from a predominantly pro-inflammatory M1-like phenotype toward an immunomodulatory M2-like phenotype within the perihematomal region, thereby potentially promoting tissue repair and limiting secondary brain injury.

**Figure 8:**
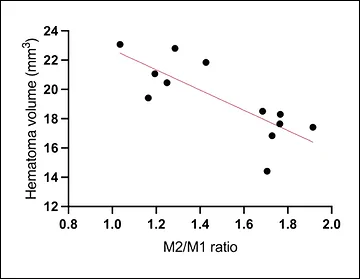
Correlation between the perihematomal M2/M1 ratio and hematoma volume in Zone 2. Linear regression analysis demonstrated a significant inverse association between the M2/M1 ratio and hematoma volume (R² = 0.6204, p = 0.0023; n = 12). ICH = Intracerebral hemorrhage; M1 = Classically activated microglia; M2 = Alternatively activated microglia; Zone 2 = Perihematomal region.

To further evaluate the relationship between microglial polarization and hematoma burden, a linear regression analysis was performed to assess the association between the perihematomal M2/M1 ratio and hematoma volume (Figure 8). A significant inverse correlation was observed (R² = 0.6204, p = 0.0023; n = 12), indicating that higher M2/M1 ratios were associated with smaller hematoma volumes. These findings further support the association between PAP-1-induced M2 polarization and reduced hemorrhagic injury following ICH.

### Neurological recovery

**Figure 9:**
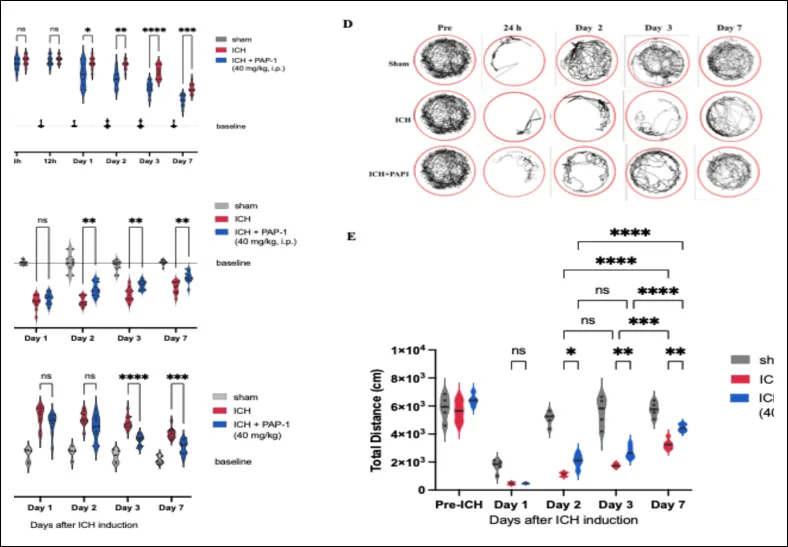
PAP-1 treatment accelerated neurological recovery following ICH. Behavioral outcomes were assessed longitudinally in Cohort 1 (n = 10 mice per group). (A) mNSS values in the Sham + vehicle, ICH + vehicle, and ICH + PAP-1 groups. The Sham + vehicle group exhibited negligible neurological deficits throughout the study. The ICH + vehicle group showed a rapid increase in mNSS at 6 h after surgery, with scores peaking at 24 h and declining gradually thereafter. PAP-1-treated mice exhibited significantly lower scores at 24 h and accelerated recovery through Day 7 (treatment effect: ω² = 0.58). (B) Cylinder test assessment of contralateral forelimb use during rearing. The Sham + vehicle group maintained consistently high performance, whereas the ICH + vehicle group exhibited marked deficits from 24 h through Day 7, indicating increased reliance on the ipsilateral forelimb. PAP-1-treated mice showed partial but significant improvement in contralateral forelimb use beginning on Day 2 (treatment effect: ω² = 0.84). (C) Corner test assessment of turning bias. The Sham + vehicle group remained near baseline, whereas the ICH + vehicle group exhibited a persistent right-turn bias following ICH induction. PAP-1 treatment progressively reduced the right-turn bias through Day 7 (treatment effect: ω² = 0.30). (D) Representative OFT movement traces for each group at baseline and on post-ICH days 1, 2, and 7. The ICH + vehicle group exhibited reduced exploratory activity, whereas the ICH + PAP-1 group showed partial recovery by Day 7. (E) Quantification of the total distance traveled during the OFT. The Sham + vehicle group maintained stable locomotor activity throughout the study. The ICH + vehicle group exhibited a marked reduction in locomotor activity at 24 h that persisted through Day 7, despite partial spontaneous recovery. Locomotor activity in the ICH + PAP-1 group did not differ from that in the ICH + vehicle group at 24 h but improved significantly beginning on Day 2 and continued to recover through Day 7. Data are presented as mean ± SEM.

Consistent with the observed anti-inflammatory and neuroprotective effects, PAP-1 administration significantly improved neurological recovery following ICH across multiple behavioral assessments (Figures 9A–E).

Two-way ANOVA demonstrated a substantial treatment effect on mNSS (ω² = 0.58). The ICH + vehicle group exhibited a marked increase in neurological deficit scores within 24 h after ICH induction (12.20 ± 0.36; p < 0.0001 versus Sham + vehicle; 95% CI = −13.05 to −10.95), indicating severe acute neurological injury. Although scores subsequently declined, recovery remained incomplete on day 7 (7.30 ± 0.30; p < 0.0001 versus Sham + vehicle; 95% CI = −8.248 to −6.152). In contrast, PAP-1-treated mice exhibited significantly lower mNSS values at 24 h (10.00 ± 0.72; p = 0.0404 versus ICH + vehicle; 95% CI = 1.152–3.248) and more rapid recovery, with scores approaching baseline by day 7 (5.00 ± 0.33; p = 0.0002 versus ICH + vehicle; 95% CI = 1.252–3.348) (Figure 9A).

The cylinder test similarly demonstrated a substantial treatment effect (ω² = 0.84). Mice in the ICH + vehicle group exhibited markedly reduced contralateral forelimb use at 24 h after ICH induction (19.70% ± 1.70%; p < 0.0001 versus Sham + vehicle), with only limited recovery by day 7 (30.67% ± 1.50%; p < 0.0001 versus Sham + vehicle). PAP-1 treatment significantly improved contralateral forelimb use beginning on day 2 (29.53% ± 1.69%; p = 0.012 versus ICH + vehicle), with continued improvement through day 7 (38.44% ± 1.43%; p = 0.0039 versus ICH + vehicle) (Figure 9B). These findings indicate that PAP-1 improved forelimb symmetry and sensorimotor recovery following ICH.

The corner test also demonstrated a significant treatment effect (ω² = 0.30). Vehicle-treated ICH mice exhibited a pronounced turning bias toward the ipsilateral right side at 24 h (83.69% ± 2.60%; p < 0.0001 versus Sham + vehicle), which persisted through day 7 (70.11% ± 1.33%; p < 0.0001 versus Sham + vehicle). No significant difference was detected between the ICH + vehicle and ICH + PAP-1 groups at 24 h (p = 0.0645). However, PAP-1 treatment significantly reduced the ipsilateral turning bias on day 3 (65.01% ± 1.32%; p < 0.0001 versus ICH + vehicle; 95% CI = 7.112–20.26) and day 7 (59.10% ± 1.88%; p = 0.0002 versus ICH + vehicle; 95% CI = 4.847–18.00) (Figure 9C). These results indicate improved preservation and recovery of contralateral sensorimotor function following PAP-1 treatment.

The OFT further supported the neurological benefits of PAP-1. Two-way ANOVA demonstrated a significant treatment effect on the total distance traveled (ω² = 0.20). In the ICH + vehicle group, the total distance traveled was significantly lower than that in the Sham + vehicle group at 24 h (0.473 ± 0.044 × 10³ cm; p = 0.0007; 95% CI = 481.8–2009) and on day 2 (1.118 ± 0.068 × 10³ cm; p < 0.0001; 95% CI = 3218–4745). Although locomotor activity gradually increased on days 3 and 7, indicating partial spontaneous recovery, it remained significantly lower than that in the Sham + vehicle group on day 3 (1.763 ± 0.061 × 10³ cm; p < 0.0001; 95% CI = 3080–4607) and day 7 (3.286 ± 0.159 × 10³ cm; p < 0.0001; 95% CI = 1707–3234).

PAP-1-treated mice showed no significant improvement in locomotor activity at 24 h compared with the ICH + vehicle group (0.482 ± 0.027 × 10³ cm; p = 0.9996; 95% CI = −772.30 to 754.70). However, significant improvement was observed beginning on Day 2 (2.078 ± 0.220 × 10³ cm; p = 0.0103; 95% CI = −1723 to −196.00), with further recovery on Day 3 (2.901 ± 0.296 × 10³ cm; p = 0.0087; 95% CI = −1741 to −214.50) and Day 7 (4.459 ± 0.111 × 10³ cm; p = 0.0014; 95% CI = −1936 to −409.40) (Figures 9D and E). Representative movement traces also showed improved exploratory activity in PAP-1-treated mice. Although locomotor activity remained lower than that in the Sham + vehicle group at the corresponding time points, PAP-1 attenuated acute neurological dysfunction, accelerated functional recovery beginning on Day 2, and improved motor and exploratory behavior following ICH.

ICH = Intracerebral hemorrhage; mNSS = Modified neurological severity score; OFT = Open field test; PAP-1 = 5-(4-phenoxybutoxy) psoralen; SEM = Standard error of the mean. *p < 0.05, **p < 0.01, ***p < 0.001 versus ICH + vehicle; ***p < 0.001 and ****p < 0.0001 versus Sham + vehicle, as applicable.

Collectively, the findings demonstrate that PAP-1, a selective Kv1.3 inhibitor, improved survival and body weight recovery, accelerated hematoma resolution, and increased neuronal survival, as reflected by greater NeuN-positive cell density. PAP-1 also reduced the accumulation of Kv1.3-positive and CD16/32-positive pro-inflammatory microglia while increasing the M2/M1 microglial phenotype ratio, indicating a shift toward an immunomodulatory and reparative response. These histological and cellular changes were accompanied by improved behavioral outcomes, including lower mNSS values, increased locomotor activity in the OFT, reduced ipsilateral turning bias in the corner test, and improved contralateral forelimb use in the cylinder test. Together, these results indicate that pharmacological Kv1.3 inhibition with PAP-1 exerted neuroprotective effects and promoted functional recovery following ICH.

## DISCUSSION

### Kv1.3 inhibition attenuates secondary brain injury and promotes hematoma resolution

Pharmacological inhibition of Kv1.3 reduced hematoma volume, improved neurological recovery, and was associated with a higher survival rate in a collagenase-induced mouse model of sICH. These findings suggest that suppression of Kv1.3 activity may not only limit hemorrhage-associated tissue injury but also attenuate the secondary pathological processes that contribute to disease progression [31]. Immunohistochemical analysis demonstrated that PAP-1 significantly reduced the accumulation of Kv1.3-positive and CD16/32-positive pro-inflammatory M1-like microglia in the perihematomal region while increasing the number of CD206-positive M2-like microglia and the M2/M1 ratio. This shift away from a predominantly pro-inflammatory microenvironment was accompanied by greater neuronal preservation, reduced tissue injury, and improved sensorimotor recovery compared with vehicle-treated sICH mice. Collectively, these findings identify Kv1.3 as a promising therapeutic target to attenuate post-sICH neuroinflammation and promote structural and functional recovery.

By selectively inhibiting an ion channel involved in microglial activation, PAP-1 may interrupt the self-perpetuating cycle of inflammation-mediated cellular injury and subsequent inflammatory amplification following sICH. This secondary injury cascade contributes to progressive neuronal death, perihematomal tissue damage, edema, and delayed neurological dysfunction during the hours and days after hemorrhage induction. Consistent with this temporal course, systemic PAP-1 administration significantly reduced hematoma volume beginning on Day 2 after sICH induction, with a progressively greater difference relative to the vehicle-treated group by Day 7. This pattern suggests that Kv1.3 blockade may accelerate hematoma resolution rather than prevent the initial hemorrhagic event.

One possible mechanism underlying the reduction in hematoma volume is enhanced clearance of erythrocytes, cellular debris, and other hemorrhagic products through expansion of the anti-inflammatory and phagocytic M2-like microglial/macrophage population [20]. This interpretation is supported by the early increase in CD206-positive cells beginning on Day 1 after sICH. Previous experimental studies have similarly shown that promotion of M2-like polarization and a shift away from M1-like predominance can facilitate hematoma resolution and improve neurological outcomes following ICH [8, 30]. Therefore, the progressive reduction in hematoma volume during PAP-1 treatment may reflect enhanced clearance of hemorrhagic debris, attenuation of secondary hematoma expansion, or both, thereby contributing to preservation of the perihematomal tissue and improvement of neurological function.

The temporal relationship between increased CD206-positive microglia and reduced hematoma volume further supports a possible role for enhanced hematoma clearance in the neuroprotective effects of PAP-1. However, this proposed mechanism remains inferential because the present study did not directly assess erythrophagocytosis, phagocytic activity, or hematoma composition. Alternative mechanisms may also have contributed, including improved vascular stability, attenuation of secondary bleeding, modulation of inflammatory edema, and altered hematoma evolution. Furthermore, because hematoma volume was not evaluated at earlier time points, such as 6–12 h after sICH induction, the study could not distinguish among effects on initial hematoma formation, secondary hematoma expansion, and subsequent hematoma clearance.

Co-localization of Kv1.3 with microglial markers, such as Iba1, was also not assessed. Therefore, although Kv1.3 immunoreactivity increased markedly in the perihematomal region, its definitive cellular localization could not be established. Further studies incorporating co-localization analyses, cell-specific Kv1.3 manipulation, and direct measurements of microglial phagocytic activity are needed to clarify the mechanisms through which PAP-1 influences hematoma evolution.

### Kv1.3 inhibition modulates microglial polarization

Kv1.3 channels have emerged as important regulators of microglial activation and provide a mechanistic link between ion channel activity and the neuroinflammatory processes that contribute to secondary brain injury after ICH [32, 33]. In the present study, Kv1.3-positive cell numbers increased markedly within perihematomal Zone 2 after sICH, reached a maximum on Day 3, and subsequently declined. This temporal and spatial pattern closely paralleled the increase in CD16/32-positive M1-like microglia, suggesting an association between Kv1.3 expression and the development of a pro-inflammatory microglial phenotype.

PAP-1 suppressed the expansion of Kv1.3-positive and CD16/32-positive cells across the evaluated regions, with the most pronounced effect observed in perihematomal Zone 2. By Day 7, the number of CD16/32-positive cells in this region had declined toward baseline in PAP-1-treated mice. Kv1.3 has been reported to regulate Ca²⁺-dependent NF-κB signaling and activation of the NLRP3 inflammasome [34, 35]. Therefore, the present findings support the role of Kv1.3 as a potential amplifier of pro-inflammatory microglial activation after sICH. By attenuating these signaling pathways, PAP-1 may suppress the propagation of inflammation from the perihematomal region and protect vulnerable but potentially salvageable neurons surrounding the hematoma.

The effects of PAP-1 extended beyond suppression of M1-like activation. Kv1.3 inhibition also altered the balance between pro-inflammatory and reparative microglial phenotypes. During the early phase after experimental sICH, microglia displayed a predominantly M1-like phenotype, characterized by increased CD16/32 expression. In contrast, CD206-positive M2-like microglia increased within the perihematomal region during the first several days after injury, consistent with their proposed roles in phagocytosis, debris clearance, angiogenesis, tissue remodeling, and neuroprotective signaling [36, 37].

A notable mechanistic finding was that PAP-1 promoted an increase in CD206-positive M2-like microglia before the peak accumulation of CD16/32-positive M1-like microglia observed in vehicle-treated mice on Day 3. The M2/M1 ratio consequently remained above 1.0 throughout the observation period in PAP-1-treated mice and increased progressively through Day 7. This temporal pattern suggests that the effects of PAP-1 involve not only suppression of inflammation but also earlier initiation of reparative processes. PAP-1 may directly promote M2-like polarization or may indirectly permit reparative microglial responses by suppressing Kv1.3-dependent pro-inflammatory signaling [38].

Nevertheless, the M1/M2 classification represents a simplified framework for describing microglial activation states. Microglial phenotypes after brain injury are heterogeneous, dynamic, and may simultaneously express markers associated with both inflammatory and reparative functions. Therefore, the observed changes in CD16/32 and CD206 should be interpreted as shifts in marker-defined activation profiles rather than definitive transitions between mutually exclusive microglial populations. Future studies incorporating single-cell transcriptomic analysis, multiplex immunophenotyping, and functional assessment of microglial activity would provide a more comprehensive understanding of the cellular responses to Kv1.3 inhibition.

### Survival and translational relevance of Kv1.3 inhibition

An important finding of the present study was the higher 7-day survival rate observed in PAP-1-treated mice compared with vehicle-treated sICH mice. Although this difference did not reach statistical significance, the numerical improvement in survival, together with reduced hematoma volume, greater neuronal preservation, and improved neurological function, supports the potential protective efficacy of Kv1.3 inhibition. However, the wide CI and limited group size indicate that the survival finding should be interpreted cautiously and confirmed in adequately powered studies.

The combined histological, immunological, and behavioral findings indicate that PAP-1 may exert neuroprotective effects by attenuating Kv1.3-associated pro-inflammatory microglial activation, promoting a more reparative microglial profile, and facilitating hematoma resolution. These changes may collectively reduce secondary neuronal injury and enhance functional recovery following sICH. Nevertheless, further studies are required to determine the optimal therapeutic window, dose–response relationship, duration of treatment, long-term efficacy, and safety of systemic Kv1.3 inhibition. Validation in female animals, aged animals, and models incorporating clinically relevant comorbidities will also be essential before the translational potential of PAP-1 can be adequately assessed.

### Kv1.3 inhibition improves functional recovery and neuronal preservation

Functional outcomes were consistently better in PAP-1-treated mice, in agreement with the histological and survival findings. Neurological deficits following ICH arise from both primary tissue injury caused by hematoma-related mechanical compression and delayed secondary injury cascades, including neuroinflammation. PAP-1 treatment significantly improved motor coordination, sensory integration, balance, and spontaneous locomotor activity compared with vehicle-treated ICH mice. These improvements may reflect preservation of critical neural circuits through attenuation of neuroinflammatory injury and promotion of reparative responses associated with a shift toward M2-like microglial predominance.

These findings are consistent with previous evidence that Kv1.3 blockade improves neurological recovery by limiting microglial-mediated toxicity [16, 39]. The behavioral improvements were further supported by the immunohistochemical findings, as PAP-1 treatment preserved significantly more NeuN-positive neurons in the perihematomal region on days 3 and 7 after ICH than vehicle treatment. This neuronal preservation may be associated with modulation of the perihematomal inflammatory response, as indicated by changes in microglial phenotype-associated markers. Consistent with this interpretation, a previous study using an ischemic stroke model demonstrated that PAP-1 reduced infarct volume and improved neurological outcomes, partly through modulation of microglial activation [31]. Thus, the present findings add to the growing evidence that targeting microglial ion channels may attenuate secondary brain injury and improve neurological function after stroke.

### Potential mechanisms underlying PAP-1-mediated neuroprotection

The neuroprotective effects associated with Kv1.3 inhibition are likely multifactorial [40]. Kv1.3 is highly expressed in activated microglia and contributes to the maintenance of their pro-inflammatory responses [41]. Inhibition of Kv1.3 has been shown to suppress microglial activation, reduce the production and release of pro-inflammatory cytokines, and limit oxidative injury in several neurological conditions [42, 43]. In the present ICH model, Kv1.3 blockade reduced the accumulation of pro-inflammatory microglia/macrophages and shifted the marker-defined phenotype toward a more reparative profile. Previous studies have similarly shown that PAP-1 can increase M2-associated markers and anti-inflammatory mediators while suppressing NF-κB pathway activation following ICH [20, 44].

Through modulation of microglial behavior, Kv1.3 inhibition may facilitate hematoma clearance by enhancing phagocytosis of erythrocytes and blood-derived debris [30] while simultaneously creating a tissue environment that favors neuronal survival [45]. In addition, attenuation of microglia-mediated inflammation may help preserve BBB integrity and reduce cerebral edema [46]. Activated microglia release TNF-α, matrix metalloproteinases, and other mediators that contribute to BBB disruption after ICH [12]. Therefore, suppression of excessive microglial activation may indirectly reduce vascular permeability, edema formation, and intracranial pressure. These effects, together with reduced oxidative stress and inflammatory cytokine toxicity, may act synergistically to improve outcomes in PAP-1-treated mice.

Kv1.3 channels regulate microglial membrane potential by maintaining the electrochemical gradient required for sustained Ca²⁺ influx. This Ca²⁺ signaling contributes to activation of downstream inflammatory pathways, including ERK1/2 and NF-κB. Therefore, inhibition of Kv1.3 by PAP-1 may attenuate these pathways, reduce the production of pro-inflammatory mediators, and limit neuroinflammation following ICH [47]. However, the present study did not directly investigate downstream signaling events associated with Kv1.3 inhibition, including intracellular Ca²⁺ dynamics, membrane potential changes, ERK1/2 signaling, NF-κB activation, or NLRP3 inflammasome activity. Accordingly, although PAP-1 treatment was associated with changes in microglial/ macrophage phenotype-associated markers, the findings do not establish a direct causal relationship between Kv1.3 blockade and microglial polarization. Further molecular and electrophysiological studies are required to define the downstream mechanisms underlying PAP-1-associated neuroprotection.

### Comparison with previous experimental ICH models

The present findings are generally consistent with those of a recent study evaluating PAP-1 in an autologous blood injection model of ICH [20]. However, differences between the studies may reflect the distinct injury kinetics of the experimental models and the temporal windows used for outcome assessment. In the autologous blood injection model, a fixed volume of blood is directly introduced into the brain parenchyma, producing an immediate hematoma. Although this model is useful for investigating secondary injury processes such as inflammation, gliosis, and tissue remodeling, it does not reproduce the progressive vascular disruption that occurs during the early phase of sICH [48].

In contrast, the collagenase-induced ICH model more closely reproduces the evolving vascular injury characteristic of spontaneous hemorrhage. Collagenase degrades the vascular basal lamina and extracellular matrix, resulting in progressive disruption of cerebral microvessels and continued bleeding. Consequently, this model produces gradual hematoma formation and ongoing vascular damage, thereby reflecting important early pathological features of sICH [24]. In the present study, outcomes were assessed during the first 7 days after ICH, a critical period characterized by vascular disruption, active hematoma evolution, perihematomal neuronal injury, and robust neuroinflammatory responses. Evaluation during this early interval is particularly relevant for investigating secondary brain injury and therapies directed at early inflammatory signaling and microglial activation.

PAP-1 produced measurable effects as early as days 2 and 3, with benefits persisting through Day 7. These effects were observed across multiple outcomes, including attenuation of body weight loss, reduction in hematoma volume, preservation of perihematomal neurons, improvement in neurological function, and a numerically higher survival rate. This early therapeutic profile suggests that PAP-1 may influence several pathological processes during the acute-to-subacute phase after ICH, including hematoma evolution, vascular instability, inflammatory amplification, and perihematomal neuronal injury.

Previous work by Wang *et al.* demonstrated beneficial effects of PAP-1 in an autologous blood injection model of ICH [20]. The present study extends those observations to a collagenase-induced model that more closely reflects progressive vascular disruption and evolving hematoma formation. To our knowledge, this is the first study to evaluate selective Kv1.3 inhibition with PAP-1 in a collagenase-induced ICH model. Within this context, PAP-1 treatment was associated with an early increase in M2-associated microglial/macrophage markers beginning on Day 1, a significant reduction in hematoma volume from Day 2, preservation of perihematomal neurons, and accelerated neurological recovery. These findings suggest that Kv1.3 inhibition may affect not only secondary neuroinflammatory mechanisms but also pathological events occurring during the early evolving phase of hemorrhagic injury.

A detailed comparison of the major methodological and outcome differences between the collagenase-induced model used in the present study and the autologous blood injection model reported by Wang *et al.* [20] is presented in Table 1. Although direct quantitative comparisons are limited by differences in experimental design, injury kinetics, treatment protocols, and outcome measures, both studies support Kv1.3 as a promising therapeutic target for modulating post-hemorrhagic neuroinflammation and improving neurological outcomes following experimental ICH.

**Table 1:** Comparison of experimental ICH models and the therapeutic effects of PAP-1 between the present study and Wang et al. [20].

**Feature**	**Present study (Collagenase-induced ICH model)**	**Wang ** ***et al.*** ** [20] (Autologous blood injection ICH model)**
ICH induction method	Enzymatic induction by intracerebral bacterial collagenase injection	Mechanical induction by intracerebral autologous blood injection
Pathophysiological characteristics	Progressive vascular degradation with ongoing bleeding and evolving hematoma formation	Immediate formation of a fixed volume hematoma producing mass effect
Experimental focus	Early vascular injury, hematoma evolution, and acute neuroinflammatory responses	Secondary brain injury, white matter damage, and subacute neuroinflammation
Kv1.3 inhibitor	PAP-1	PAP-1
Dose	40 mg/kg	40 mg/kg
Observation period	6 h to Day 7 after ICH	Primarily subacute and chronic phases (up to Day 7 or longer)
Neuroprotective effects	Reduced hematoma volume, preservation of perihematomal neurons, attenuation of body weight loss, and numerically improved survival	Attenuation of white matter injury and improvement of neurological deficits
Microglial modulation	Increased CD206-positive M2-associated microglia/macrophages, reduced Kv1.3-positive and CD16/32-positive M1-associated microglia, and increased M2/M1 ratio	Modulation of M1/M2 microglial polarization through suppression of the NF-κB signaling pathway
Hematoma outcome	Significant reduction in hematoma volume beginning on Day 2 after ICH	Significant reduction in hematoma volume reported on Day 7
Behavioral outcomes	Improved mNSS, Cylinder test, Corner test, and OFT performance	Improved OFT performance and BMS scores
Translational relevance	More closely reproduces progressive vascular injury, hematoma evolution, and early pathological events characteristic of spontaneous ICH	Primarily suitable for investigating secondary injury mechanisms after establishment of a fixed hematoma
Overall interpretation	Suggests that Kv1.3 inhibition influences both early hemorrhagic evolution and secondary neuroinflammatory responses	Demonstrates the anti-inflammatory and neuroprotective effects of Kv1.3 inhibition during the secondary injury phase

BMS = Basso Mouse Scale; CD = Cluster of differentiation; ICH = Intracerebral hemorrhage; Kv1.3 = Voltage-gated potassium channel Kv1.3; M1 = Classically activated microglia; M2 = Alternatively activated microglia; mNSS = Modified neurological severity score; NF-κB = Nuclear factor-kappa B; OFT = Open field test; PAP-1 = 5-(4-phenoxybutoxy) psoralen.

### Association between microglial phenotype and hematoma burden

The inverse association between the perihematomal M2/M1 ratio and hematoma volume provides additional support for a possible relationship between PAP-1-induced modulation of microglial phenotype and hematoma resolution. Although correlation analysis cannot establish causality, the findings indicate that higher M2/M1 ratios were associated with lower hematoma volumes during the early phase after collagenase-induced ICH.

M2-associated microglia/macrophages participate in the clearance of erythrocytes, cellular debris, and other blood-derived components following hemorrhage. Therefore, the observed shift toward an M2-dominant marker profile may contribute to more efficient removal of hematoma contents and attenuation of secondary inflammatory injury. The timing of this relationship is notable because it occurred while hematoma evolution and neuroinflammatory responses were actively progressing, further supporting the potential relevance of Kv1.3 inhibition during the acute phase of sICH.

Nevertheless, the reduction in hematoma volume should not be attributed exclusively to enhanced M2-associated phagocytic clearance. Other mechanisms may also contribute, including reduced secondary bleeding, improved vascular stability, attenuation of perihematomal edema, altered erythrocyte lysis, and changes in the temporal evolution of the hematoma. Because direct measurements of erythrophagocytosis, vascular integrity, BBB function, and hematoma degradation were not performed, the relative contribution of these mechanisms remains uncertain. Future studies incorporating these assessments are needed to clarify how Kv1.3 inhibition influences hematoma evolution and resolution.

### Strengths, limitations, and future directions

The present study extends the therapeutic relevance of Kv1.3 modulation from previously investigated ischemic and neurodegenerative conditions to hemorrhagic stroke. A principal strength was the use of a collagenase-induced ICH model, which enabled evaluation of PAP-1 during progressive vascular injury and evolving hematoma formation. The study also integrated survival, body weight, histological, immunohisto-chemical, and behavioral outcomes, thereby providing a comprehensive assessment of structural and functional responses to Kv1.3 inhibition during the acute and subacute phases of ICH.

Several limitations should nevertheless be acknowledged. First, although the collagenase-induced ICH model is widely used and highly reproducible, it does not reproduce all features of human sICH. Collagenase causes enzymatic disruption of cerebral vessels, which can produce progressive hemorrhage and a prolonged inflammatory response that may be more severe than those observed in many patients with spontaneous ICH [49]. This may limit direct translation of the findings. Future studies should therefore validate Kv1.3 inhibition in complementary models, including autologous blood injection and other approaches that more closely reproduce a single hemorrhagic event [2].

Second, hematoma evolution was not evaluated at ultra-early time points, such as 6–12 h after induction. Therefore, the study could not determine whether PAP-1 influenced initial hematoma formation, secondary hematoma expansion, or subsequent hematoma clearance. Future studies should include early serial hematoma quantification, brain hemoglobin measurements, vascular permeability assays, and analyses of vascular integrity to distinguish these potential effects.

Third, although PAP-1 is considered a relatively selective Kv1.3 inhibitor, off-target activity involving other potassium channels or unrelated molecular targets cannot be entirely excluded. Consequently, the observed benefits cannot be attributed exclusively to Kv1.3 inhibition. Studies using microglia-specific Kv1.3 knockdown or knockout models, together with comparisons among structurally distinct Kv1.3 inhibitors, would strengthen target-specific mechanistic conclusions.

Fourth, PAP-1 treatment was initiated within 1 h after ICH induction. This early intervention protocol may not reflect the treatment window available in clinical practice. Delayed-treatment studies, including administration at 4–6 h or later after ICH, are required to determine whether Kv1.3 inhibition remains effective within a clinically relevant therapeutic window.

Fifth, the immunohistochemical markers used in the present study did not distinguish resident microglia from infiltrating peripheral macrophages because CD16/32 and CD206 can be expressed by both populations. Therefore, the observed changes in M1- and M2-associated markers may represent the combined response of resident and infiltrating myeloid cells. Moreover, the M1/M2 framework provides only a simplified representation of the heterogeneous and dynamic activation states adopted by microglia and macrophages after brain injury. Future studies should use cell-specific markers, flow cytometry, lineage-tracing approaches, multiplex immuno-phenotyping, and single-cell transcriptomic analysis to define the cellular populations affected by PAP-1 more precisely.

Sixth, inflammatory cytokines and phenotype-associated mediators were not directly quantified. Key pro- and anti-inflammatory markers, including IL-1β, TNF-α, IL-10, and Arg1, were not evaluated at the transcriptional or protein levels. Apoptosis-related signaling, BBB integrity, and immunofluorescence co-localization of Kv1.3 with microglial/macrophage markers were also not examined. These limitations restrict mechanistic interpretation of the relationship between Kv1.3 inhibition, inflammatory signaling, and microglial phenotype modulation. Future studies incorporating quantitative polymerase chain reaction, enzyme-linked immunosorbent assay, multiplex cytokine profiling, Western blotting, and high-resolution co-localization analysis are warranted.

Seventh, only male mice were used. This approach reduced potential variability associated with sex hormone fluctuations during the initial mechanistic investigation; however, sex-related differences in microglial function, inflammatory signaling, and ICH outcomes are well established. Studies incorporating female animals and sex-balanced experimental designs are therefore required to determine whether PAP-1 has comparable efficacy across sexes.

Eighth, although PAP-1-treated mice had a numerically higher 7-day survival rate and a lower estimated mortality risk than vehicle-treated ICH mice, the pairwise survival difference was not statistically significant, and the CI for the HR crossed unity. The study was not specifically powered to detect survival differences as a primary outcome. Therefore, the apparent survival benefit should be considered exploratory and requires confirmation in larger, adequately powered studies.

Finally, the study focused on acute and subacute outcomes through Day 7. The long-term effects of Kv1.3 inhibition on functional recovery, neurogenesis, synaptic plasticity, white matter integrity, glial scar formation, brain remodeling, and chronic neuroinflammation were not assessed. It remains uncertain whether the early benefits of PAP-1 persist during the chronic phase of ICH. Future studies should include longer follow-up periods, delayed-treatment paradigms, multiple dosing regimens, female cohorts, and molecular validation of downstream signaling pathways to define the durability, safety, and translational potential of Kv1.3-targeted immunomodulation following sICH.

## CONCLUSION

The present study demonstrates that pharmacological inhibition of Kv1.3 with PAP-1 confers significant neuroprotective effects in a collagenase-induced mouse model of sICH. PAP-1 treatment was associated with attenuation of hematoma volume, preservation of perihematomal neurons, accelerated recovery of body weight, and improved neurological function across multiple behavioral assessments. These structural and functional improvements were accompanied by marked suppression of Kv1.3-positive and CD16/32-positive M1-associated microglia/macrophages, enhancement of CD206-positive M2-associated microglia/ macrophages, and a sustained increase in the M2/M1 ratio within the perihematomal region. Furthermore, the significant inverse association between the M2/M1 ratio and hematoma volume supports a potential relationship between microglial phenotype modulation and improved hematoma resolution following Kv1.3 inhibition. Although survival was numerically higher in the PAP-1-treated group, this finding should be interpreted cautiously because statistical significance was not achieved.

From a practical perspective, these findings identify Kv1.3 as a promising therapeutic target for limiting secondary brain injury during the early phase of sICH. Unlike therapies directed solely at hematoma evacuation or symptomatic management, selective Kv1.3 inhibition offers an immunomodulatory strategy that simultaneously suppresses detrimental neuroinflammation while promoting reparative microglial responses. The demonstration of therapeutic efficacy in a collagenase-induced model, which closely reproduces the progressive vascular injury and evolving hematoma characteristic of sICH, further strengthens the translational relevance of this approach. These findings also provide a rationale for future studies evaluating Kv1.3 inhibition in clinically relevant treatment windows, diverse experimental models, both sexes, and larger preclinical studies incorporating detailed molecular analyses and long-term functional outcomes.

In conclusion, selective Kv1.3 inhibition with PAP-1 shifted the perihematomal inflammatory response toward a reparative phenotype, reduced hematoma burden, preserved neuronal integrity, and accelerated neurological recovery following experimental sICH. Collectively, these findings provide compelling preclinical evidence that Kv1.3 represents a promising therapeutic target for modulating post-hemorrhagic neuroinfla-mmation and improving functional recovery. Further mechanistic investigations and translational studies are warranted to establish the safety, therapeutic window, and long-term efficacy of Kv1.3-targeted immuno-modulation before clinical application in patients with sICH.

## DATA AVAILABILITY

The datasets generated and analyzed during the present study are available from the corresponding author upon reasonable request.

## GENERATIVE AI DECLARATION

The authors used generative artificial intelligence (AI) solely to improve the language, grammar, and readability of the manuscript. The authors carefully reviewed and edited all AI-assisted output and take full responsibility for the content of this manuscript. AI was not used to generate research data, analyze data, interpret results, or draw scientific conclusions. AI is not listed as an author.

## AUTHORS’ CONTRIBUTIONS

WS: Conceptualization, methodology, formal analysis, and writing-original draft. NT and AV: Methodology, data collection, and histological analysis. AW and YB: Methodology, technical support, and provision of experimental resources. WW and ST: Conceptualization, methodology, supervision, and visualization. NP: Conceptualization, funding acquisition, project administration, supervision, and writing-review and editing. All authors have read and approved the final version of the manuscript.
